# Protective Effect of Wheat Peptides against Indomethacin-Induced Oxidative Stress in IEC-6 Cells

**DOI:** 10.3390/nu6020564

**Published:** 2014-01-29

**Authors:** Hong Yin, Xingchang Pan, Zhixiu Song, Shaokang Wang, Ligang Yang, Guiju Sun

**Affiliations:** 1 Key Laboratory of Environmental Medicine and Engineering of Ministry of Education, Department of Nutrition and Food Hygiene, School of Public Health, Southeast University, Nanjing 210009, China; E-Mails: 230119540@seu.edu.cn (H.Y.); 230079359@seu.edu.cn (S.W.); 230119541@seu.edu.cn (L.Y.); 2 China National Research Institute of Food & Fermentation Industries, Beijing 100028, China; E-Mail: 2008107031@njau.edu.cn; 3 Second School of Clinical Medical, Nanjing University of Chinese Medicine, Nanjing 210023, China; E-Mail: 230099500@seu.edu.cn

**Keywords:** wheat peptides, indomethacin, IEC-6 cells, oxidative stress, NF-κB-iNOS-NO signal pathway

## Abstract

Recent studies have demonstrated that wheat peptides protected rats against non-steroidal anti-inflammatory drugs-induced small intestinal epithelial cells damage, but the mechanism of action is unclear. In the present study, an indomethacin-induced oxidative stress model was used to investigate the effect of wheat peptides on the nuclear factor-κB(NF-κB)-inducible nitric oxide synthase-nitric oxide signal pathway in intestinal epithelial cells-6 cells. IEC-6 cells were treated with wheat peptides (0, 125, 500 and 2000 mg/L) for 24 h, followed by 90 mg/L indomethacin for 12 h. Wheat peptides significantly attenuated the indomethacin-induced decrease in superoxide dismutase and glutathione peroxidase activity. Wheat peptides at 2000 mg/L markedly decreased the expression of the NF-κB in response to indomethacin-induced oxidative stress. This study demonstrated that the addition of wheat peptides to a culture medium significantly inhibited the indomethacin-induced release of malondialdehyde and nitrogen monoxide, and increased antioxidant enzyme activity in IEC-6 cells, thereby providing a possible explanation for the protective effect proposed for wheat peptides in the prevention of indomethacin-induced oxidative stress in small intestinal epithelial cells.

## 1. Introduction

Traditional non-steroidal anti-inflammatory drugs (NSAIDs) like aspirin and indomethacin are widely used for the relief of pain and inflammation. However, their use is limited by their gastrointestinal toxicity [1,2]. NSAIDs promote reactive oxygen species (ROS) production. It has been proposed that NSAID-mediated gastrointestinal lesions involve the uncoupling of oxidative phosphorylation and inhibition of the electron transport chain causing incomplete reduction of oxygen. Indomethacin, a potent NSAID, was found to bind to a site near complex I and ubiquinone to generate ROS [3,4]. ROS can damage cellular lipids, proteins, and DNA leading to oxidative stress [5]. ROS can damage mitochondrial aconitase, which leads to the release of iron from its iron-sulfur cluster. This free iron reacts with H_2_O_2_, producing hydroxyl radical, which amplifies the oxidative stress [6]. Oxidative stress-induced functional loss is well correlated with the severity of small intestinal mucosal lesions [7,8]. NF-κB plays a central role in the induction of small intestinal mucosal cell damage during NSAID-induced oxidative stress. It has been documented that NF-κB has been identified as a transcription factor regulated by the intracellular redox status, which is activated by oxidative stress and induces the expression of a variety of proteins such as inducible nitric oxide synthase (iNOS) and cyclo-oxygenase-2 (COX-2) in small intestinal mucosal cells during NSAID treatment [9,10].

Wheat peptides belong to a family of biologically active peptide derived from wheat protein. These peptides have been found to act as antioxidant agonists under *in vivo* and *in vitro* conditions [11,12]. So antioxidant therapy would be a realistic approach to prevent NSAID-induced oxidative damage.

Our last thesis showed that the treatment with wheat peptides could cause potential effects on epithelial cell of small intestine by decreasing in oxidative stress conditions in rats [13], but its mechanism was unclear.

Presented here, the aims of this study were to investigate whether management with wheat peptides has any potential effects and possible signal pathway in small intestinal epithelial cell of NSAID-induced oxidative damage.

## 2. Materials and Methods

### 2.1. Materials

Indomethacin and 3-(4,5-dimethyl-2-yl)-2,5-diphenyltetrazolium bromide (MTT) were from Sigma Chemical Co. (St. Louis, MO, USA). Fetal bovine serum (FBS) was from ICN Biomedicals Inc. (Costa Mesa, CA, USA). Dulbecco’s modified Eagles medium (DMEM) were from GIBCO BRL (Rockville, MD, USA). All other chemicals were the highest purity available in the market.

Wheat peptides were obtained from China National Research Institute of Food & Fermentation Industries, Beijing, China. The molecular weight was 140–1000 Da, which was obtained by the protease hydrolysis method. These peptides from 140 to 1000 Da account for 92% of the total weight of all the prepared peptides. Wheat peptides in this study almost included 3–6 amino-acid residues [14].

### 2.2. Cell Culture

IEC-6 cells (rat jejunal crypt cell line) were purchased from Institute of Zoology, Chinese Academy of Sciences (Kunming, China) and cultured in Dulbecco’s modified Eagle medium High Glucose (DMEM), supplemented with 10% fetal bovine serum (FBS), 100 IU/mL penicillin, and 100 IU/mL streptomycin in a humidified incubator at 95% relative humidity and 5% CO_2_ at 37 °C.

### 2.3. Cell Survival Assays

Cell survival was assessed by using the 3-(4,5-dimethyl-2-yl)-2,5-diphenyltetrazolium bromide (MTT). IEC-6 cells were plated and incubated in a 96-well plate for 3 days. Then various concentrations of indomethacin (0, 5.625, 11.25, 22.5, 45, and 90 mg/L) were added to the wells. After 12 h, 20 µL of the MTT solution (0.5 mg/mL PBS) was added to each well, and the cells were incubated for 4 h at 37 °C. Then 100 µL of dimethyl sulfoxide (DMSO) were added and thoroughly mixed. Optical density at 490 nm was measured using a microplate reader. For comparative purpose, the data were converted to percentage of control.

IEC-6 cells were plated and incubated for 2 days in a 96-well plate. Then various concentrations of wheat peptides (0, 125, 500 and 2000 mg/L) were added to the wells. After 24 h, 90 mg/L indomethacin was added to each well. After 12 h, 20 µL of the MTT solution (0.5 mg/mL PBS) was added to each well, and the cells were incubated for 4 h at 37 °C. Then 100 µL of dimethyl sulfoxide (DMSO) were added and thoroughly mixed. Optical density at 490 nm was measured using a microplate reader. For comparative purposes, the data were converted to percentage of control.

### 2.4. Treatment

IEC-6 cells were grown in six-well plates (1 × 10^6^ cells/well) for 2 days and treated with 0, 125, 500 or 2000 mg/L wheat peptides or vehicle for 24 h. After this, 90 mg/L indomethacin was added to each well for 12 h (Table 1). Following the incubation period, the cells were harvested.

**Table 1:** Treatment of the wheat peptides and indomethacin in IEC-6 cells.

	Control	M Control	L	M	H
Wheat peptides (mg/L), 24 h	0	0	125	500	2000
Indomethacin (mg/L), 12 h	0	90	90	90	90

L = Low dose wheat peptides treated (125 mg/L); M = moderate dose wheat peptides treated (500 mg/L); H = High dose wheat peptides treated (2000 mg/L).

### 2.5. Assays of MDA, SOD, GPx, Catalase in the Supernatant of IEC-6 Cells

Superoxide Dismutase (SOD), Glutathione peroxidase (GPx), catalase (CAT) activity and malondialdehyde (MDA) concentration were determined using commercial kits that had been purchased from the Nanjing Jiancheng Bioengineering Institute (Nanjing, China), following the manufacturer’s instructions.

### 2.6. Western Blot Analysis

IEC-6 cells, cultured on six-well plates, were washed twice in 2 mL ice-cold phosphate buffer saline (PBS), and the cells were collected into an eppendorf tube (gently scraped by a rubber policeman) and centrifuged at 1000 rpm for 5 min at 4 °C. Cell membranes were lysed by incubating on ice for 30 min with lysis buffer (Beyotime, Nantong, China). The supernatants were collected by centrifugation at 1500 rpm for 10 min at 4 °C, protein concentration was determined by bicinchoninic acid (BCA) assay and stored at −70 °C until used.

Cellular proteins were separated by 10% sodium dodecyl sulfate-polyacrylamide gel electrophoresis (SDS-PAGE) and transferred onto Poly(vinylidene fluoride) transfer membranes (Millipore, Bedford, MA, USA). Membranes were blocked for 3 h with 5% non-fat milk in TBST and then incubated overnight with a rabbit polyclonal antibody against NF-κB p65 (dilution 1:1500, Santa Cruz, CA, USA). After washing with Tris Buffer Solution Tween (TBST) for three times, membranes were incubated with horseradish peroxidase-conjugated secondary antibodies for 1 h at room temperature (dilution 1:5000, Boster Co., Wuhan, China). The signal was detected by Electro-Chemi-Luminescence (ECL) superSignalTM West Pico substrate (Pierce, Rockford, IL, USA). Mouse anti-Lamin B1 monoclonal antibody (Bioworld Technology Inc., Nanjing, China) was used as the loading control, and NF-κB p65 protein expression was normalized to Lamin B1.

### 2.7. Assays of iNOS, NO in the Supernatant of IEC-6 Cells

The inducible nitric oxide synthase (iNOS) activity was determined using an enzyme linked immunosorbent assay (ELISA) kit that had been purchased from Science Biotechnology Co. Ltd. (Yantai, China), following the manufacturer’s instructions.

The nitric oxide (NO) concentration was determined using commercial kits that had been purchased from the Applygen Technologies (Beijing, China), following the manufacturer’s instructions.

### 2.8. Statistical Analyses

All experiments were carried out in triplicate, and the data were presented as the mean ± standard error (SEM). Differences considered significant at *p* < 0.05 were determined by *t*-test for independent samples or one-way analysis of variance (ANOVA) performed using statistical software SPSS13.0 (StatSoft, Tulsa, OK, USA).

## 3. Results

### 3.1. Changes in Survival of IEC-6 Cells after Indomethacin Treatment

**Figure 1:**
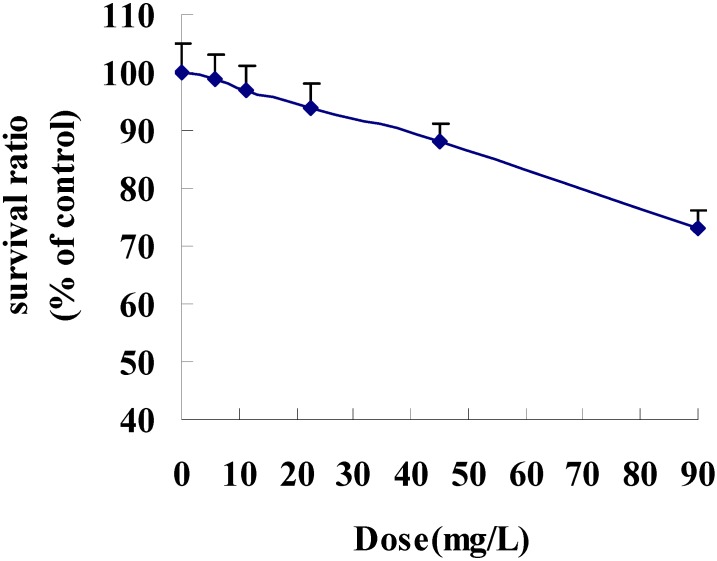
Effect of indomethacin on survival in IEC-6 cells; Data are presented as the means ± SE (*n* = 5).

As shown in Figure 1, indomethacin induced a dose-dependent decrease in survival ratio (5.625, 11.25, 22.5, 45, and 90 mg/L).

### 3.2. Changes in Survival of IEC-6 Cells after Wheat Peptides Treatment

**Figure 2:**
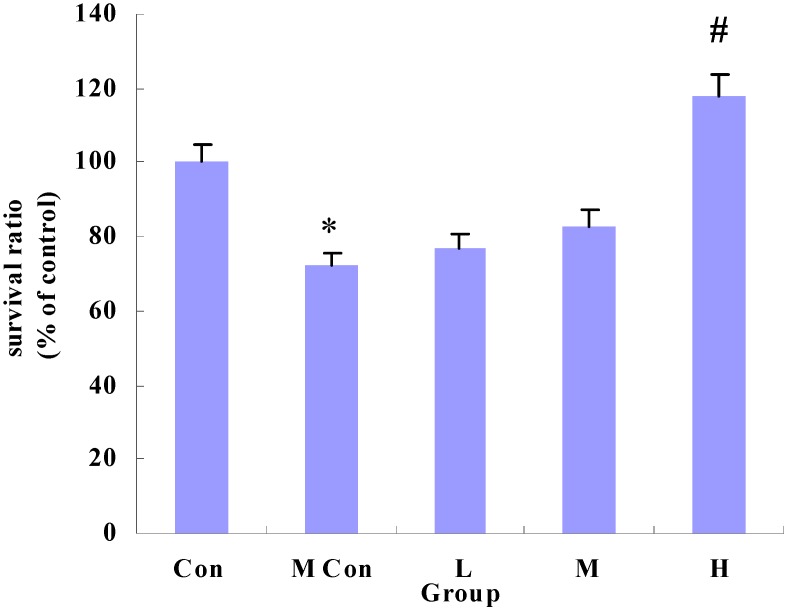
Changes in the survival ratio of IEC-6 cells after wheat peptides treatment. IEC-6 cells were pre-cultured in medium for 48 h, then treated with different doses of wheat peptides for 24 h, finally followed by indomethacin (90 mg/L) for 12 h. Data are presented as the means ± SE (*n* = 5); * *p* < 0.05 significantly different from the normal control groups; ^#^ *p* < 0.05 significantly different from the model groups (the indomethacin groups).

As shown in Figure 2, indomethacin significantly reduced survival ratio of IEC-6 cells (*p* < 0.05), while the survival ratio of IEC-6 cells was markedly increased by wheat peptides (2000 mg/L).

### 3.3. Changes in Antioxidant Activity and Malondialdehyde (MDA) Concentration of IEC-6 Cells after Wheat Peptides Treatment

As shown in Table 2, indomethacin-induced decreases in SOD and GPx activity were markedly increased by wheat peptides compared to the model groups, while indomethacin-induced MDA concentration significantly increased in IEC-6 cells (*p* < 0.05).

**Table 2:** Activities of enzymatic antioxidants such as superoxide dismutase (SOD), catalase and Glutathione peroxidase (GPx) and levels of malondialdehyde (MDA) of IEC-6 cells.

	SOD(U/mL)	Catalase (U/mL)	GPx(U/mL)	MDA(nmol/mL)
Control	20.97 ± 0.14	6.64 ± 0.55	65.32 ± 1.14	5.6 ± 0.56
Model Control	17.09 ± 0.22 *	5.76 ± 0.48 *	65.32 ± 4.56	8.8 ± 0.91 *
L	18.13 ± 0.18	5.67 ± 0.50	72.56 ± 0.57 ^#^	7.6 ± 0.69
M	17.38 ± 0.13	5.67 ± 0.51	71.35 ± 1.14 ^#^	6.8 ± 0.56
H	18.99 ± 0.22 ^#^	5.95 ± 0.66	70.95 ± 0.57 ^#^	6.4 ± 0.58 ^#^

Results are expressed as the mean ± S.D. (*n* = 3); * Significantly different from the control group (*p* < 0.05); ^#^ Significantly different from the model control group (*p <* 0.05).

### 3.4. Changes in NF-κB-iNOS-NO of IEC-6 Cells after Wheat Peptides Treatment

**Figure 3:**
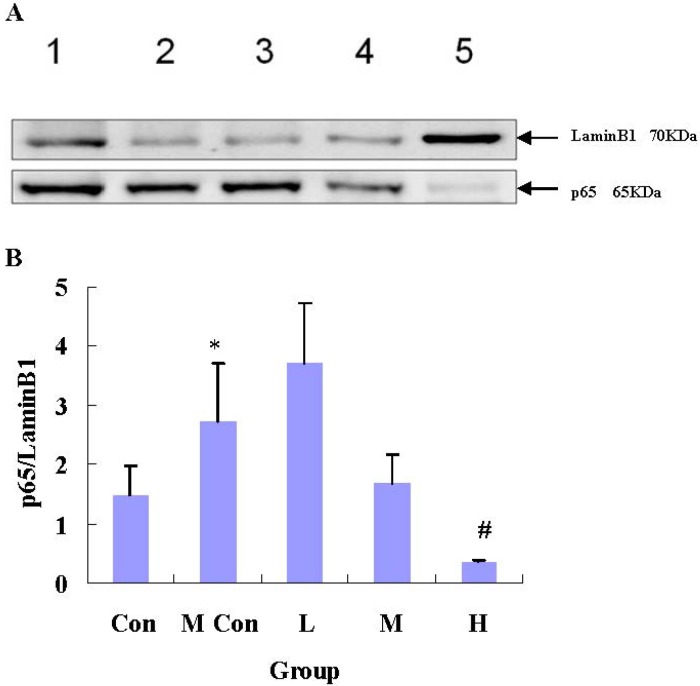
Changes in the expression of NF-κB p65 of IEC-6 cells after wheat peptides treatment; IEC-6 cells were pre-cultured in medium for 48 h, then treated with different doses of wheat peptides for 24 h, finally followed by indomethacin (90 mg/L) for 12 h. Data are presented as the means ± SE (*n* = 3); A: 1 = Con, 2 = M Con, 3 = L, 4 = M, 5 = H; * *p* < 0.05 significantly different from the normal control groups; ^#^ *p* < 0.05 significantly different from the model groups.

The level of NF-κB p65 was significantly higher in IEC-6 cells treated with 90 mg/L indomethacin than the control groups (*p* < 0.05). However, 2000 mg/L wheat peptides significantly decreased the level of p65 in IEC-6 cells (*p* < 0.05), compared with the model groups (Figure 3).

**Figure 4:**
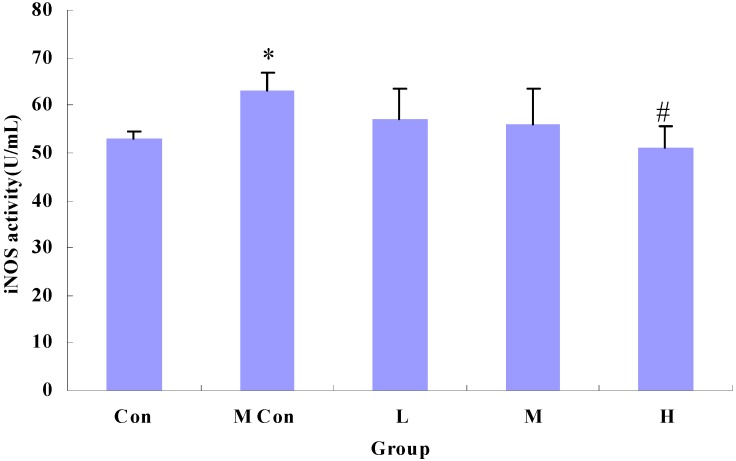
Changes in nitric oxide synthase (iNOS) activity of IEC-6 cells after wheat peptides treatment; IEC-6 cells were pre-cultured in medium for 48 h, then treated with different doses of wheat peptides for 24 h, finally followed by indomethacin (90 mg/L) for 12 h. Data are presented as the means ± SE (*n* = 3); * *p* < 0.05 significantly different from the normal control groups; ^#^ *p* < 0.05 significantly different from the model groups (the indomethacin groups).

**Figure 5:**
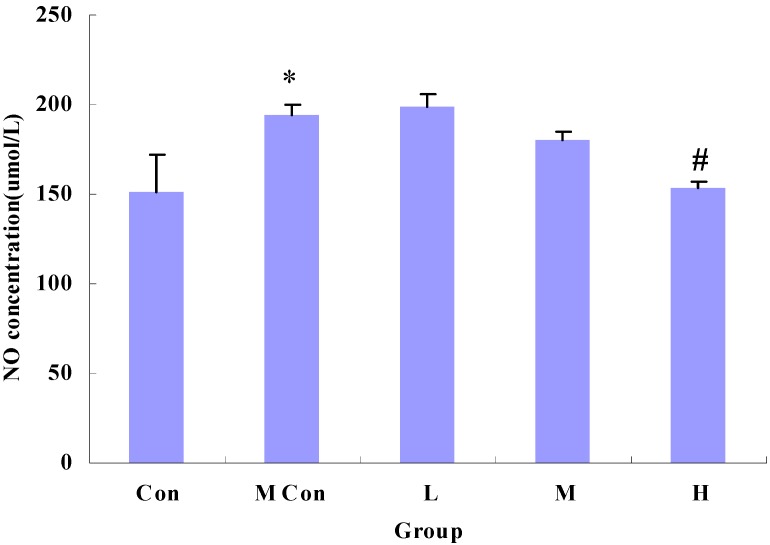
Changes in NO concentration of IEC-6 cells after wheat peptides treatment; IEC-6 cells were pre-cultured in medium for 48 h, then treated with different doses of wheat peptides for 24 h, finally followed by indomethacin (90 mg/L) for 12 h. Data are presented as the means ± SE (*n* = 3); * *p* < 0.05 significantly different from the normal control groups; ^#^ *p* < 0.05 significantly different from the model groups (the indomethacin groups).

As shown in Figure 4 and Figure 5, indomethacin-induced increases in iNOS activity and NO concentration were markedly reduced by wheat peptides compared to the model groups.

## 4. Discussion

Widespread use of NSAIDs in clinical settings to ameliorate pain and inflammation is accompanied by a range of negative side effects, most notably in the gastrointestinal tract [15]. Numerous mechanisms of action have been proposed to mediate the toxic effects of NSAIDs on GI epithelia [16], but of particular interest to our laboratory is oxidative stress by NSAIDs. Previously, there was an important observation in the case of oxidative stress mediated NSAIDs: our laboratory found that wheat peptides can exert antioxidant activity *in vivo* [13]. Therefore, we sought to determine if epithelia activity and NSAID-mediated oxidative stress were affected by treatment with wheat peptides *in vitro*, providing a possible physiological and molecular mechanism through which these wheat peptides inhibit oxidative stress in the GI tract.

Oxidative stress plays an important role in small intestinal mucosal damage. In IEC-6 cells, mitochondria are the major source of ROS and also a sensitive target for ROS-mediated damage [17,18]. Increased generation of ROS causes oxidative stress and is responsible for small intestinal mucosal injury [19]. Previously, we reported that non-steroidal anti-inflammatory drugs induced small intestinal damage in rats over 2 h. Wheat peptides administration may be an effective tool for protecting small intestinal tissue against NSAID-induced small intestinal damage and oxidative stress. The MDA levels, which indicate lipid peroxidation of the membranes, were significantly increased after indomethacin treatment, which is closely related to cell damage [20]. To find out whether the protective activity of wheat peptides is mediated through its antioxidant action, MDA was measured in IEC-6 cells in the presence or absence of wheat peptides (Table 2). Our results indicated that indomethacin-induced oxidative stress was statistically decreased by wheat peptides.

Preventive antioxidants such as SOD and catalase enzymes are the first line of defense against reactive oxygen species [21]. In our study, the administration of wheat peptides attenuated indomethacin-induced oxidative damage by increasing SOD activity. This indicates that wheat peptides protect the IEC-6 cells by preserving antioxidant enzyme activity in the mucosa exposed to damage. CAT is another enzymatic antioxidant predominantly present in peroxisomes and ameliorates the deleterious effect of hydrogen peroxide, which is produced by SOD, into water and non-reactive oxygen species [22,23]. It restrains the procreation of hydroxyl radical and protects the cells and tissues from oxidative damage. Glutathione (GSH) is an important constituent of intracellular protective mechanisms against a number of noxious stimuli, and it is known to be a major low molecular weight scavenger of free radicals in cytoplasm [24]. The GSH redox cycle catalyzed by the endogenous antioxidant enzyme GPx reduces H_2_O_2_. At the same time, the antioxidant activity of GPx is coupled with the oxidation of GSH to GSSG, which can subsequently be reduced by glutathione reductase with triphosphopyridine nucleotide (NADPH) as the reducing agent [25,26]. Our results indicated that GPx activity was statistically increased by wheat peptides. These results indicate that wheat peptides can increase antioxidant enzyme activities with good efficacy in IEC-6 cells. These results were consistent with the case of NSAIDs-induced oxidative stress *in vivo*.

NF-κB has been identified as a transcription factor regulated by the intracellular redox status, which is activated by oxidative stress and induces the expression of a variety of proteins such as cyclooxygenase (COX)-2 and iNOS that function in the immunological and cellular detoxifying defense systems [27,28]. Another humoral factor that contributes to mucosal protection during inflammatory reactions is nitric oxide (NO), which is produced by nitric oxide synthase (iNOS) in cells. However, when excessively produced, NO may react with superoxide anion radical, giving rise to the strong oxidant peroxynitrite and damaging functional tissues [29]. Therefore, a beneficial therapeutic strategy involves the inhibition of NF-κB-iNOS-NO signal pathway under oxidative stress condition. Our data shows significantly increased NO levels in IEC-6 cells under indomethacin-induced oxidative stress, which is consistent with the Nagai’s result [30]. Treatment with wheat peptides caused marked reduction in NO levels in IEC-6 cells.

Finally, we can conclude that the treatment with wheat peptides may cause potential effects such as pronounced decreasing in stress condition and inhibiting NF-κB-iNOS-NO signal pathway in NSAID-induced oxidative damage. Our results may aid in the development of new methods for enhancing the health of the small intestine.

## References

[1] Bjarnason I. (2013). Gastrointestinal safety of NSAIDs and over-the-counter analgesics. Int. J. Clin. Pract..

[2] Jarupongprapa S., Ussavasodhi P., Katchamart W. (2013). Comparison of gastrointestinal adverse effects between cyclooxygenase-2 inhibitors and non-selective, non-steroidal anti-inflammatory drugs plus proton pump inhibitors: A systematic review and meta-analysis. J. Gastroenterol..

[3] Kang H.S., Ock J., Lee H.J., Lee Y.J., Kwon B.M., Hong S.H. (2013). Early growth response protein 1 upregulation and nuclear translocation by 2’-benzoyloxycinnamaldehyde induces prostate cancer cell death. Cancer Lett..

[4] Vaish V., Piplani H., Rana C., Vaiphei K., Sanyal S.N. (2013). NSAIDs may regulate EGR-1-mediated induction of reactive oxygen species and non-steroidal anti-inflammatory drug-induced gene (NAG)-1 to initiate intrinsic pathway of apoptosis for the chemoprevention of colorectal cancer. Mol. Cell. Biochem..

[5] Droge W. (2002). Free radicals in the physiological control of cell function. Physiol. Rev..

[6] Valko M., Leibfritz D., Moncol J., Cronin M.T.D., Mazur M., Telser J. (2007). Free radicals and antioxidants in normal physiological functions and human disease. Int. J. Biochem. Cell Biol..

[7] Correa P., Houghton J. (2007). Carcinogenesis of helicobacter pylori. Gastroenterology.

[8] Odabasoglu F., Cakir A., Suleyman H., Aslan A., Bayir Y., Halici M., Kazaz C. (2006). Gastroprotective and antioxidant effects of usnic acid on indomethacin-induced gastric ulcer in rats. J. Ethnopharmacol..

[9] Webb D.L., Rudholm-Feldreich T., Gillberg L., Halim M.A., Theodorsson E., Sanger G.J., Campbell C.A., Boyce M., Naslund E., Hellstrom P.M. (2013). The type 2 CCK/gastrin receptor antagonist YF476 acutely prevents NSAID-induced gastric ulceration while increasing iNOS expression. Naunyn-Schmied. Arch. Pharmacol..

[10] Laube M., Kniess T., Pietzsch J. (2013). Radiolabeled COX-2 inhibitors for non-invasive visualization of COX-2 expression and activity—A critical update. Molecules.

[11] Cavazos A., de Mejia E.G. (2013). Identification of bioactive peptides from cereal storage proteins and their potential role in prevention of chronic diseases. Compr. Rev. Food Sci. Food Saf..

[12] Zhu K.X., Guo X., Guo X.N., Peng W., Zhou H.M. (2013). Protective effects of wheat germ protein isolate hydrolysates (WGPIH) against hydrogen peroxide-induced oxidative stress in PC12 cells. Food Res. Int..

[13] Yin H., Pan X.C., Wang S.K., Yang L.G., Sun G.J. (2014). Protective effect of wheat peptides against small intestinal damage induced by non-steroidal anti-inflammatory drugs in rats. J. Integr. Agric..

[14] Pan X., Yin H., Gu R., Xu Y., Cai M., Sun G. (2013). Effect of wheat peptide on the nitrogen metabolism and gastrointestinal mucosal structure of rats. Food Sci..

[15] Fujita T., Kutsumi H., Sanuki T., Hayakumo T., Azuma T. (2013). Adherence to the preventive strategies for nonsteroidal anti-inflammatory drug- or low-dose aspirin-induced gastrointestinal injuries. J. Gastroenterol..

[16] Satoh H., Takeuchi K. (2012). Management of NSAID/aspirin-induced small intestinal damage by GI-sparing NSAIDs, anti-ulcer drugs and food constituents. Curr. Med. Chem..

[17] Murphy M.P. (2009). How mitochondria produce reactive oxygen species. Biochem. J..

[18] Wallace D.C. (2005). A mitochondrial paradigm of metabolic and degenerative diseases, aging, and cancer: A dawn for evolutionary medicine. Annu. Rev. Genet..

[19] Omatsu T., Naito Y., Handa O., Mizushima K., Hayashi N., Qin Y., Harusato A., Hirata I., Kishimoto E., Okada H. (2010). Reactive oxygen species-quenching and anti-apoptotic effect of polaprezinc on indomethacin-induced small intestinal epithelial cell injury. J. Gastroenterol..

[20] Amir M., Shikha K. (2004). Synthesis and anti-inflammatory, analgesic, ulcerogenic and lipid peroxidation activities of some new 2-(2,6-dichloroanilino) phenyl acetic acid derivatives. Eur. J. Med. Chem..

[21] Jainu M., Devi C.S.S. (2006). Gastroprotective action of Cissus quadrangularis extract against NSAID induced gastric ulcer: Role of proinflammatory cytokines and oxidative damage. Chem.-Biol. Interact..

[22] Gonzalez-Rey M., Bebianno M.J. (2012). Does non-steroidal anti-inflammatory (NSAID) ibuprofen induce antioxidant stress and endocrine disruption in mussel Mytilus galloprovincialis?. Environ. Toxicol. Pharmacol..

[23] Parolini M., Binelli A., Provini A. (2011). Chronic effects induced by ibuprofen on the freshwater bivalve Dreissena polymorpha. Ecotoxicol. Environ. Saf..

[24] Mates J.M., Segura J.A., Alonso F.J., Marquez J. (2011). Anticancer antioxidant regulatory functions of phytochemicals. Curr. Med. Chem..

[25] De la Lastra C.A., Motilva V., Martin M.J., Nieto A., Barranco M.D., Cabeza J., Herrerias J.M. (1999). Protective effect of melatonin on indomethacin-induced gastric injury in rats. J. Pineal Res..

[26] Kasperska-Zajac A., Brzoza Z., Rogala B., Polaniak R., Birkner E. (2008). Antioxidant enzyme activity and malondialdehyde concentration in the plasma and erythrocytes of patients with urticaria induced by nonsteroidal anti-inflammatory drugs. J. Investig. Allergol. Clin. Immunol..

[27] Hussein S.Z., Yusoff K.M., Makpol S., Yusof Y.A.M. (2012). Gelam honey inhibits the production of proinflammatory, mediators NO, PGE(2), TNF-alpha, and IL-6 in carrageenan-induced acute paw edema in rats. Evid.-Based Complement. Altern. Med..

[28] Takeuchi K., Tanaka A., Kato S., Amagase K., Satoh H. (2010). Roles of COX inhibition in pathogenesis of NSAID-induced small intestinal damage. Clin. Chim. Acta.

[29] Mikulec C.D., Rundhaug J.E., Simper M.S., Lubet R.A., Fischer S.M. (2013). The chemopreventive efficacies of nonsteroidal anti-inflammatory drugs: The relationship of short-term biomarkers to long-term skin tumor outcome. Cancer Prev. Res..

[30] Nagai N., Takeda A., Itanami Y., Ito Y. (2012). Co-administration of water containing magnesium ion prevents loxoprofen-induced lesions in gastric mucosa of adjuvant-induced arthritis rat. Biol. Pharm. Bull..

